# Hamartomatous polyposis syndromes: A review

**DOI:** 10.1186/1750-1172-9-101

**Published:** 2014-07-15

**Authors:** Anne Marie Jelsig, Niels Qvist, Klaus Brusgaard, Claus Buhl Nielsen, Tine Plato Hansen, Lilian Bomme Ousager

**Affiliations:** 1 Department of Clinical Genetics, Odense University Hospital and Institute of Clinical Research, University of Southern Denmark, Sdr. Boulevard 29, 5000 Odense C, Denmark; 2 Surgical Department A, Odense University Hospital Denmark, Sdr. Boulevard 29, 5000 Odense C, Denmark; 3 Department of Clinical Genetics, Odense University Hospital Denmark, Sdr. Boulevard 29, 5000 Odense C, Denmark; 4 Department of Surgery, Hvidovre Hospital, Kettegaard Alle 30, 2650 Hvidovre, Denmark; 5 Department of Pathology, Odense University Hospital, Sdr. Boulevard 29, 5000 Odense C, Denmark

**Keywords:** Hamartomatous polyposis syndromes, PTEN hamartoma tumor syndrome, Peutz-Jeghers syndrome, Juvenile polyposis syndrome, neurofibromatosis type 1, Gorlin Syndrome, Juvenile polyp, Polyposis

## Abstract

Hamartomatous Polyposis Syndromes (HPS) are genetic syndromes, which include Peutz-Jeghers syndrome, Juvenile polyposis syndrome, *PTEN* hamartoma tumour syndrome (Cowden Syndrom, Bannayan-Riley-Ruvalcaba and Proteus Syndrome) as well as hereditary mixed polyposis syndrome. Other syndromes such as Gorlin Syndrome and multiple endocrine neoplasia syndrome 2B are sometimes referred to as HPS. HPS is characterized by the development of hamartomatous polyps in the gastrointestinal tract as well as several extra-intestinal findings such as dermatological and dysmorphic features or extra-intestinal cancer. The syndromes are rare and inherited in an autosomal dominant manner.

The diagnosis of HPS has traditionally been based on clinical criteria, but can sometimes be difficult as the severity of symptoms range considerably from only a few symptoms to very severe cases - even within the same family. *De novo* cases are also frequent. However, because of the discovery of several associated germline-mutations as well as the rapid development in genetics it is now possible to use genetic testing more often in the diagnostic process. Management of the syndromes is different for each syndrome as extra-intestinal symptoms and types of cancers differs.

Clinical awareness and early diagnosis of HPS is important, as affected patients and at-risk family members should be offered genetic counselling and surveillance. Surveillance in children with HPS might prevent or detect intestinal or extra-intestinal complications, whereas in adulthood surveillance is recommended due to an increased risk of cancer e.g. intestinal cancer or breast cancer.

## Introduction

Hamartomatous Polyposis Syndromes (HPS) are rare genetic syndromes characterized by the development of hamartomatous polyps in the gastrointestinal tract (GI-tract). Despite variable phenotypic expression of the syndromes affected patients have an increased risk of cancer and surveillance is relevant from an early age. The aim of this article is to give an updated review of clinical features, genetics, treatment and surveillance of HPS.

## Methods

This review is based on a literature search using PubMed and Medline including original articles, reviews, cases and clinical guidelines. The search terms were “hamartomatous polyposis syndromes”, “Peutz-Jeghers syndrome”, “juvenile polyposis syndrome”, “Peutz-Jeghers polyp”, “juvenile polyp”, “*PTEN* hamartoma tumour syndrome” (Cowden syndrome, Bananyan-Riley-Ruvalcaba), “hereditary mixed polyposis syndrome”, “Neurofibromatosis type 1”, “Gorlin Syndrome” and “multiple endocrine neoplasia syndrome 2B”. *Inclusion- and exclusion criteria:* Case reports, retrospective cohort studies and papers discussing guidelines for therapy, surveillance, phenotype or genetics were included. Reviews were mostly used to search the bibliography to identity additional papers of interest. When newer papers were based on or discussing older papers e.g. surveillance strategy the newest paper was included. Each paper, which was identified in the search, was reviewed in order to determine suitability. Only English language articles up to January 2014 were included.

## Background

Hamartomatous polyps in the GI-tract are rare compared to neoplastic and hyperplastic polyps. However, the hamartomatous polyp is the most common type of polyps in children [1]. The prevalence in the entire population is unknown but some suggest a prevalence of approximately 2% in the paediatric population [2].

**Figure 1:**
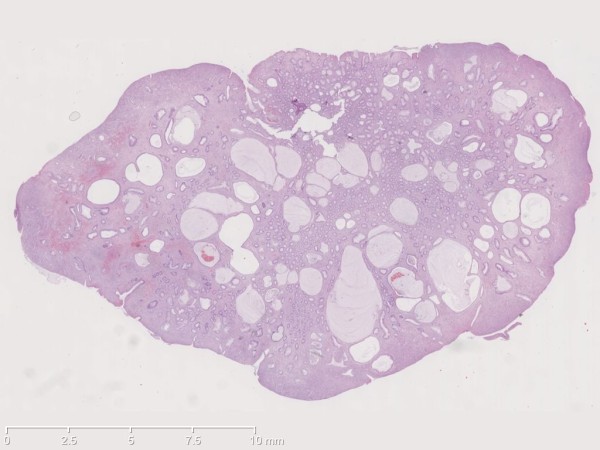
Histopathology of a juvenile polyp with characteristic cystic glands.

**Figure 2:**
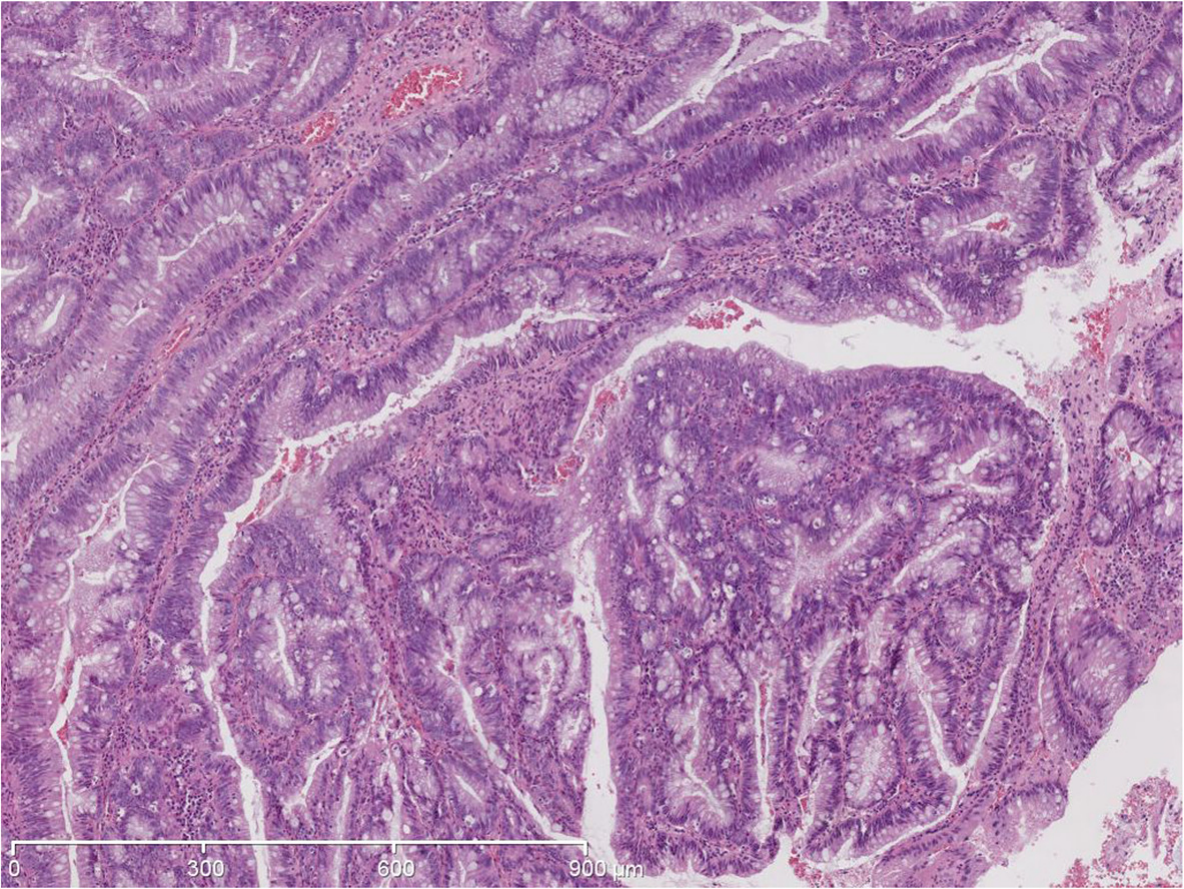
Histopathology of a juvenile polyp with dysplasia.

Hamartomatous polyps are most often diagnosed by endoscopy and symptoms include rectal bleeding, pain, anaemia, prolapsing polyp, diarrhoea and/or melena [3]. The hamartomatous polyps vary in size and may have a characteristic histological structure, which makes it possible to distinguish between the *Peutz-Jeghers polyp* and the *juvenile polyp.* Peutz-Jeghers polyps are typically multilobulated with a papillary surface and branching bands of smooth muscle covered by hyperplastic glandular mucosa [4]. The term “*juvenile polyps”* refer to a special histopathology and not the age of onset as the polyp might be diagnosed at all ages. The juvenile polyp has a spherical appearance and is microscopically characterized by overgrowth of an oedematous lamina propria with inflammatory cells and cystic glands (Figures 1 and 2). It might be difficult the distinguish between an inflammatory and a juvenile polyp [2].

Solitary hamartomatous polyps in the gastrointestinal tract is generally not considered to be associated with an increased risk of cancer [5], but >2 polyps and/or a family history of colorectal cancer or polyps should lead to suspicion of HPS*.* In addition to the occurrence of polyps, many of syndromes also manifest with extra-intestinal findings such as developmental delay, dermatological abnormalities or extra-intestinal cancer. HPS should be distinguished from *Adenomatous Polyposis Syndromes* such as Familiar Adenomatous Polyposis Syndrome (FAP) or MUTYH-associated Polyposis (MAP), which are dominated by the presence of adenomas.

The syndromes are inherited in an autosomal dominant pattern with offspring having a 50% risk of inheriting the condition. The expressivity of the phenotype is variable and penetrance is often 100%. During the past two decades several genes have been associated with HPS (Table 1). Detection of a germline mutation in the proband makes identification and surveillance of at-risk family members possible. Thus genetic counselling should be a cornerstone in the diagnostic approach to patients suspected for a polyposis syndrome. Furthermore affected patients and at-risk family members should be offered surveillance. The lack of long-term follow-up studies of the effect of surveillance, presumable ascertainment bias in the reported risks of cancer and the somewhat unknown pathogenic basis for cancer-development makes decision on a surveillance program challenging.

**Table 1:** Overview of the most common Hamartomatous Polyposis Syndromes

	**Genes**	**Hallmark features**	**Cancer by site**	**Approx. mutation detection rate (%)**
Juvenile polyposis syndrome	*SMAD4, BMPR1A*	Multiple GI-polyps, epistaxis,* telangiectasia*	Colon, rectum and stomach	60% [2]
PTEN-hamartoma syndrome: Cowden Syndrome	*PTEN*	Lhermitte-Duclos disease, trichilemmoma, skin hamartoma, macrocephaly,	Breast, thyroid, uterus, colon	Up to 80% [6]
PTEN-hamartoma syndrome: Bannayan-Riley-Ruvalcaba	*PTEN*	Macrocephaly, lipomatosis, pigmented macules of the glans penis	As above	60% [7]
Peutz-Jeghers syndrome	*STK11 (LKB1)*	Mucocutanous melanosis and polyposis of the GI-tract	Colon, stomach, breast, pancreas (cervix, ovarian)	80%-94% [8]
Hereditary mixed polyposis syndrome	*(BMPR1A, GREM1)*	Atypical polyposis with juvenile polyps, adenomas, hyperplastic and inflammatory	Colon and rectum	Unknown

*In *SMAD4* mutation carriers.

HPS include several syndromes of which the following are discussed in this article: *Juvenile polyposis syndrome, PTEN-hamartoma tumor syndrome, Peutz-Jeghers syndrome, and Hereditary mixed polyposis syndrome*.

### Juvenile polyposis syndrome

Juvenile polyposis syndrome (JPS) is characterized by the occurrence of multiple juvenile polyps in the GI-tract. The incidence is approximately between 1:100.000 to1:160 000 [3]. The number of polyps varies from 1 to over a 100, and may be found in the entire GI-tract but mostly in the colon, rectum and ventricle. The age at diagnosis varies considerably, but symptoms usually present themselves in the first and second decade [9].

The diagnostic criteria for JPS was established in 1975 and later revised by *Jass et al.*[10]. According to these one of the following must be present:

1) More than five juvenile polyps in the colorectum or/and

2) Multiple juvenile polyps throughout the GI-tract or/and

3) Any number of juvenile polyps with a family history of juvenile polyposis

JPS is inherited in an autosomal dominant manner and 20-50% of affected patients have a positive family history [9]. In patients fulfilling the diagnostic criteria it is possible to detect mutations in *BMPR1A* in 20-30% of patients, and in *SMAD4* in 20-30% of the patients. Both genes are part of the transforming growth factor (TGF-beta) pathway.

An increased lifetime risk for both colorectal cancer (CRC) and gastric cancer has been documented in several studies: *Howe et al.* reported a 38% lifetime risk of developing CRC and 21% for upper GI cancer [11]. *Brosens et al.* reported a relative risk of developing CRC of 34.0 with the cumulative lifetime risk of 38.7% [12]. Median age at diagnosis was in the latter study 42 years. Pancreatic cancer, and cancer of the small intestine has been reported in a small number of patients with JPS [11,13]. Reports suggest that *SMAD4*-mutations carriers have a significant higher frequency of gastric polyps and gastric cancer than *BMPR1A*-mutation carriers [14,15].

There is no international consensus on treatment or prophylactic surgery for patients. For some patients endoscopic polypectomies will be sufficient. Prophylactic total – or subtotal colectomy or gastrectomy should be considered in patients with multiple polyps, severe symptoms or a family history of CRC [16-18]. Proctocolectomy and subtotal colectomy with ileorectal anastomosis need endoscopic follow-up because of high recurrence-rate of polyps [16].

For asymptomatic at-risk members of JPS-families (e.g. mutation-positive individuals) British recommendations from 2009–10 suggest surveillance with colonoscopy every 1–2 years from age 15–18 years until age 70 and gastroduodenoscopy from age 25 with a 1–2 year interval [18]. *Howe et al.* suggested that mutation carriers and asymptomatic patients should have colonoscopy every three years from age 15 years, while patients who have polyps should be examined every year [17]. They also suggested gastroduodenoscopy to be performed from age 15. *Latchford et al.* concluded that that small-bowel disease is not a significant clinical problem in JPS and as such surveillance of the small intestines should not be performed in all patients [3].

Patients with S*MAD4* mutations should be screened for hereditary hemorrhagic telangiectasia, (HHT) symptoms, in particular the presence of pulmonary AV-malformations. *McDonald et al*. published guidelines for managing HHT patients [19]. A recent report described the presence of thoracic aortic dilatation in a *SMAD4*-mutation carrier and the authors suggested that the thoracic aorta should be screened in these patients [20].

*BMPR1A* is located in the same chromosomal region as *PTEN* and larger deletions involving both genes have been reported [21]. These patients present with a severe form of JPS with onset in early childhood (sometimes called Juvenile polyposis of infancy) or might have symptoms of both Cowden syndrome and JPS.

### *PTEN*-hamartoma Tumour syndrome

*PTEN*-hamartoma Tumour syndrome include the clinical entities of Cowden syndrome (CS), Bannayan-Riley-Ruvalcaba (BRRS), *PTEN*-related Proteus syndrome (PS), and Proteus-like syndrome.

### Cowden syndrome

CS is characterized by overgrowth of multiple hamartomas involving several organs. The incidence is thought to be approximately 1:200 000, but the expressivity of the syndrome varies greatly [6]. Because Cowden syndrome (CS) is likely underdiagnosed, the actual proportion of *de novo* cases cannot be determined. CS is inherited in an autosomal dominant manner and mutations may be found in *PTEN*. The mutations are typically pointmutations or smaller deletions or insertions. The mutation-detection rate in patients fulfilling the diagnostic criteria has been reported to 80% [6] in earlier reports, however the mutation-detection rate might be lower as suggested by *Pilarksi et al.*[22].

The clinical features of CS involve benign mucocutaneous lesions and Lhermitte-Duclos Disease (LDD). LDD is dysplatic cerebellar gangliocytoma and mucocutaneous lesions include trichilemmomas, acral keratoses and papillomatous lesions. Macrocephaly is a hallmark feature and has been reported in 84% of CS patients with *PTEN*-mutations [23]. In the third decade of life more than 90% of CS-patients have symptoms. International diagnostic clinical criteria were established in 2000 [24] and have been revised over the years. In a recent article *Pilarski et al.* found that e.g. benign breast disease or genitourinary malformations could not be used as diagnostic criteria, whereas e.g. autism spectrum disorders, colon cancer and esophageal glycogenic acanthosis could be included [22]. The clinical criteria of the PTEN-hamartoma tumor syndrome as suggested by *Pilarski et al*. can be seen in Table 2.

**Table 2:** **Clinical criteria for *PTEN *** **hamartoma tumor syndrome as suggested by *Pilarski et al.*** [22]

**Major criteria**	
•	Breast cancer
•	Endometrial cancer (epithelial)
•	Thyroid cancer (follicular)
•	Gastrointestinal hamartomas (including ganglioneuromas, but excluding hyperplastic polyps; ≥3)
•	Lhermitte-Duclos disease (adult)
•	Macrocephaly (≥97 percentile: 58 cm for females, 60 cm for males)
•	Macular pigmentation of the glans penis
•	Multiple mucocutaneous lesions (any of the following)
	o Multiple trichilemmomas (≥3, at least one biopsy proven)
	o Acral keratoses (≥3 palmoplantar keratotic pits and/or acral hyperkeratotic papules)
	o Mucocutaneous neuromas (≥3)
	o Oral papillomas (particularly on tongue and gingiva), multiple (≥3) OR biopsy proven OR dermatologist diagnosed
**Minor criteria**	
•	Autism spectrum disorder
•	Colon cancer
•	Esophageal glycogenic acanthosis (≥3)
•	Lipomas (≥3)
•	Mental retardation (ie, IQ ≤ 75)
•	Renal cell carcinoma
•	Testicular lipomatosis
•	Thyroid cancer (papillary or follicular variant of papillary)
•	Thyroid structural lesions (eg, adenoma, multinodular goiter)
•	Vascular anomalies (including multiple intracranial developmental venous anomalies)
**Operational diagnosis in an individual (either of the following)**	
1. Three or more major criteria, but one must include macrocephaly, Lhermitte-Duclos disease, or gastrointestinal hamartomas; or	
2. Two major and three minor criteria.	
**Operational diagnosis in a family where one individual meets revised *PTEN *** **hamartoma tumor syndrome clinical diagnostic criteria or has a *PTEN *** **mutation:**	
1. Any two major criteria with or without minor criteria; or	
2. One major and two minor criteria; or	
3. Three minor criteria.	

The increased risk of cancer in CS-patients is well described and involves especially the thyroid (non-medullary) and breast. *Bubien et al*. [25] calculated standardised incidence ratios (SIR) for several cancer-sites in a cohort of *PTEN*-mutation positive patients. The authors found SIR for breast cancer to be 39.1, thyroid cancer 43.2 (female) and 199.5 (male), melanoma 28.3 (female) and 39.4 (male) and endometrial cancer 48.7. The cumulative risk of cancer was at the age of 70 years estimated to 85% for any cancer, 77% for breast-cancer in females and 38% for thyroid cancer (20). *Tan et al.*[26] reported a life-time risk of breast cancer to be 85.2%, thyroid cancer 35.2%, endometrial cancer 28.2%, colorectal cancer 9.0%, renal carcinoma 33.6% and melanoma 6%. The study included 368 *PTEN*-mutations positive individuals (295 index patients and 73 relatives identified following screening) and thus the high risk-estimates might be an ascertainment bias.

Hamartomatous polyps are the most common abnormal finding in the GI-tract in CS-patients [27] and have been reported in 35-85% of patients [28]. Ganglioneuromatous polyps, colonic lipomas and other types of polyps such as hyperplastic polyps, adenomas or inflammatory polyps might also be present. *Heald et al.* found that 50.4% (n = 62) of 127 patients with *PTEN*-mutations had GI-polyps and 24 patients had both upper and lower GI-polyps [28]. *Stanich et al.*[29] reported that 9 out of 10 patients meeting the diagnostic criteria had GI-polyps, with 7 patients having more than one polyp.

Surveillance of the breast, thyroid and endometrial cancer is indicated and the increased risk of colorectal cancer, renal cell carcinoma and possibly melanoma should be taken into consideration. *Tan et al.* suggests that *PTEN-*mutation carriers under the age of 18 years should undergo annual targeted history and physical examination (including dermatologic examination, neurological and psychological testing) as well as baseline thyroid examination with ultrasound [26]. From age 30 women should have an annual mammogram and an annual endometrial sampling or transvaginal ultrasound. From age 40 both men and woman should have biannual colonoscopy and biannual renal ultrasound. For patients with a heavy polyp burden more frequent colonoscopy should be considered [26]. Guidelines from the National Comprehensive Cancer Network (NCCN) are listed in Table 3. NCCN also emphasizes addressing the risk for relatives, genetic counseling and discussion of reproductive options [30].

**Table 3:** **Management program for men and woman with CS from the National Comprehensive Cancer Network**[30]

**Woman**	
•	Breast awareness starting at age 18 y
•	Clinical breast exam, every 6–12 month, starting at age 25 y or 5–10 y before the earliest known breast cancer in the family
•	Annual mammography and breast MRI screening starting at age 30–35 y or individualized based on earliest age of onset in family
•	For endometrial cancer screening, encourage patient education and prompt response to symptoms and participation in a clinical trial to determine the effectiveness or necessity of screening modalities
•	Discuss risk-reducing mastectomy and hysterectomy and counsel regarding degree of protection, extent of cancer risk and reconstruction options
•	Address psychosocial, social, and quality-of-life aspects of undergoing risk-reducing mastectomy and/or hysterectomy
**Men and Woman**	
•	Annual comprehensive physical exam starting at age 18 y or 5 y before the youngest age of diagnosis of a component cancer in the family (whichever comes first), with particular attention to breast and thyroid exam
•	Annual thyroid starting at age 18 y or 5-10 y before the earliest known thyroid cancer in the family, whichever is earlier
•	Colonoscopy, starting at age 35 y, then every 5 y or more frequently if patient is symptomatic or polyps found
•	Consider renal ultrasound starting at age 40 y, then every 1-2 y
•	Dermatological management may be indicated for some patients
•	Consider psychomotor assessment in children at diagnosis and brain MRI if there are symptoms
•	Education regarding the signs and symptoms of cancer

Adapted with permission from the NCCN Clinical Practice Guidelines in Oncology (NCCN Guidelines(r)) for Genetic/Familial High-Risk Assessment: Breast and Ovarian V.1.2014. (c) 2014 National Comprehensive Cancer Network, Inc. All rights reserved. The NCCN Guidelines(r) and illustrations herein may not be reproduced in any form for any purpose without the express written permission of the NCCN. To view the most recent and complete version of the NCCN Guidelines, go online to NCCN.org. NATIONAL COMPREHENSIVE CANCER NETWORK(r), NCCN(r), NCCN GUIDELINES(r), and all other NCCN Content are trademarks owned by the National Comprehensive Cancer Network, Inc.

### Bannayan-Riley-Ruvalcaba

Bannayan-Riley-Ruvalcaba syndrome is a part of the clinical spectrum caused by *PTEN*-mutations. *Marsch et al.* found that 60% of 43 patients with clinical BRRS had germline mutations in *PTEN*[7].

BRRS is characterized by macrocephaly (as in CS), lipomatosis and pigmented macules of the glans penis. Other features are high birth weight, hamartomatous intestinal polyposis and intellectual disability [23]. Multiple juvenile polyps have been reported in about 25% of cases [31]. Screening recommendations have not been established for BRRS but BRRS patients with *PTEN*-mutations should undergo the same surveillance programs as patients with CS.

### PTEN-related Proteus syndrome and Proteus-like-syndrome

*PTEN*-related Proteus syndrome (PS) and Proteus-like syndrome are traditionally placed under the *PTEN*-hamartoma syndrome. The syndromes are characterized by disproportionate overgrowth of affected tissues and may affect the skeleton, skin and central nervous system. The clinical presentations of the syndromes vary greatly.

*Lindhurst et al*. found that PS is caused by a somatic activation mutation in *AKT1*[32]*.* Although cases of patients with PS or Proteus-like-syndrome with *PTEN*-germline mutation have been described [33,34] some has questioned the clinical diagnosis of these patients [35,36]. Nevertheless, both *ATK1* and *PTEN* work in the PI3KCA/AKT - pathway. Treatment and surveillance of PS is based on experience from reported cases and is symptomatic, but the complications and symptoms of PS are often severe. *Turner et al.*[36] suggested periodic ophthalmologic evaluations and that baseline-brain MRI (because of CNS-complications) and educational intervention (because of cognitive impairment) could be considered. The association between deep vein thrombosis and PS has been reported several times and *Turner et al*. [36] concluded that pulmonary embolism is a major contributor to early mortality in PS. However, there is still no evidence or data to support recommendation of anticoagulation therapy.

### Peutz-Jeghers syndrome

Peutz-Jeghers syndrome (PJS) is characterized by mucocutanous melanosis, polyposis of the GI-tract, luminal gastrointestinal cancer and extraintestinal cancer. The incidence has been estimated to be approximately 1:8300 and 1:200.000 [37]. About 55% of patients have an affected parent.

**Figure 3:**
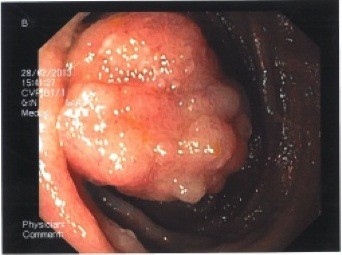
Peutz-Jeghers polyp found at endoscopy.

Patients often present themselves in infancy or early childhood with rectal bleeding, intestinal invagination, anemia and mucocutaneous pigmentations. The latter are present in approximately 95% of patients and occur on the lips, buccal mucosa and the perianal area. The lesions might fade after puberty but may persist on the buccal mucosa [8]. The number of polyps might vary from one to hundreds and are located throughout the GI-tract although mostly in the small intestines and colon [8] (Figure 3). The polyps reveal a characteristic histopathology as described earlier: The polyps have convoluted, elongated glands and an arborizing pattern of growth and consist of a branching framework of smooth muscle and connective tissue lined by normal epithelium [38]. The polyps may also be found extra-intestinally e.g. in the gall bladder, the bronchi, the urinary bladder and the ureter [39].

PJS is inherited in an autosomal dominant manner. Germline mutations can be found in *STK11* and include point-mutations as well as larger deletions. *STK11* is a tumour suppressor gene affecting the mTOR pathway. Mutations can be found in up to 94% of patients who fulfil the diagnostic criteria [40].

Diagnosis is based on the clinical presentation, the characteristic histopathology of the polyps, the results of genetic testing and family history. The diagnosis can be made when one of the following criteria is present:

1. Two or more histologically confirmed Peutz-Jeghers polyps

2. Any number of PJ-polyps detected in one individual who has a family history of PJS within close relative(s).

3. Characteristic mucocutaneous pigmentation in an individual who has a family history of PJS within close relative(s).

4. Any number of PJ-polyps in an individual who also has characteristic mucocutaneous pigmentations.

The increased risk of cancer in the GI-tract as well as extra-intestinal cancer in patients with PJS is well documented and high (Table 4). *Resta et al.* investigated the prevalence and risk of cancer in a retrospective cohort study of 119 patients who either fulfilled the diagnostic criteria or had a mutation in *STK11*[41]*.* The most frequent sites of malignancy were the GI-tract and the breast. *Giardiello et al*. conducted a meta-analysis of 210 individuals described in six publications. A statistically high significant RR for both GI-cancer and extra intestinal cancer was observed, but no significant RR for testicular or cervical cancer [42] (Table 4); the overall cumulative risk for cancer was over 90% in patients with PJS. *Van Lier et al.* concluded that PJS patients already at a young age carry a high cancer risk and the authors found the average age at cancer-diagnosis (not site-specific) to be 42 years [43]. The cumulative risk of cancer increases with age in several studies [41,44].

**Table 4:** Relative risk of cancer in Peutz-Jeghers syndrome

	***Resta et al. *** [41]	***Giardiello et al. *** [42]
**Study design**	**Retrospective cohort study**	**Meta-analysis**
Any cancer	15.1 (CI 10.5-21.2)	15.2
Breast	12.5 (CI 5.1-26.0)	15.2
Cervix	55.6 (CI 17.7-134.0)	1.5
Gynaecological cancers	27.7 (CI 11.3-57.6)	NA
Colorectal cancer	13.5 (CI 4.3-32-5)	84.0 (Colon)
Pancreas	139.7 (CI 61.1-276.4)	132
Gastrointestinal cancer	126.2 (CI 73.3-203.4)	NA
Ovary	NA	27.0
Uterus	NA	16.0
Testes	NA	4.5
Lung	NA	17.0
Small intestines	NA	520.0
Stomach	NA	213.0
Oesophagus	NA	57.0

Surveillance is essential because of the risk of complications to polyps such as intussusception as well as the increased risk of cancer. Especially surveillance of the breast, colon and rectum and the small intestines should be established. Different guidelines and recommendations have been suggested through the past decade [8,37,38,45], however, *Beggs et al*. [8] compared the surveillance programs described in the literature. The authors advocate to postpone the endoscopic screening of the GI-tract until late childhood/early adolescence and suggest that a baseline colonoscopy and upper gastroscopy is performed at age 8 years, or earlier if the patient is symptomatic, and repeated every third year if polyps are detected [8]. Otherwise the screening program should begin at age 18 and then performed every 3 years. At the age of 50 years the frequency of screening is suggested to increase to every 1–2 years because of the age-depending cancer risk. *Beggs et al.* suggested that small bowel screening should be initiated at age 8 and performed every 3 years if polyps were found or else from age 18. *Korsse et al.* discussed radiologic and endoscopic imaging modalities of the small bowel and suggested small bowel surveillance in PJS patients every 2–3 years from age 8–10 (40). *Korsse et al*. also stated that the removal of small bowel hamartomas reduces the need for elective and emergency laparotomy and that when significant polyps are detected (10–15 mm) push enteroscopy should be the preferred and polyps removed [46].

Routine-screening of pancreas has recently been investigated and the authors found that surveillance should only be performed in defined research protocols as surveillance has not yet proven to reduce mortality [47].

As part of the Dutch recommendations for surveillance *Van lier et al*. suggested that patients with a positive family history were screened with physical examination including testicular palpation and haemoglobin analysis from the age of 10 years [43]. Breast examination and breast MRI should start from 25 years of age. Colonoscopy should start at age 25–30 years with an interval of 2–5 years depending on findings (38).

### Hereditary mixed polyposis syndrome

Hereditary mixed polyposis syndrome (HMPS) was described by *Whitelaw et al*. in a large family with atypical polyps and an autosomal dominant pattern of inheritance [48]. The condition is characterized by an atypical pattern of polyposis in the colon and rectum. Affected individuals may develop juvenile polyps, hyperplastic polyps or adenomas. Colorectal carcinoma occurs in a high proportion of reported families [49]. The genetic cause (s) of HMPS remains elusive. HMPS has been mapped to the chromosomal region of 10q23, which includes *BMPR1A*[50] and O’*Riordan et al.* found a *BMPR1A* mutation in one family [51]. *Jaeger et al.* reported that a duplication in the 3’ end of the *SCG5* gene and a region upstream of *GREM1*-locus can cause HMPS [49].

Consensus surveillance programs have not been published and the existing literature is not sufficient to recommend or suggest frequency of surveillance. However, as *BMPR1A* mutation-carriers have a high risk of cancer in JPS, it seems reasonable to offer mutations-carriers a surveillance program with colonoscopy [51].

### Other syndromes with the presence of hamartomatous polyps

The presence of hamartomatous polyps is described in patients with other syndromes such as Neurofibromatosis type 1, Multiple Endocrine Neoplasia type 2B, Gorlin Syndrome and Birt-Hogg-Dubé. The syndromes are listed in Table 5.

**Table 5:** List of other syndromes where hamartomatous polyps are frequent

	**Clinical hallmarks**	**Gene**	**Cancer**
Gorlin syndrome	Keratocysts of the jaw, hyperkeratosis of palm and soles, basal cell carcinomas, skeleton abnormalities, macrocephaly, frontal bossing	*PTCH1*	Basal cell carcinomas, medullablastoma
Multiple endocrine neoplasia type 2B	Mucosal neuromas of the lips an tongue, medullated corneal nerve fibers, distinctive facies with enlarged lips and tongue, an asthenic “marfarnoid habitus, and medullary thyroid cancer.	*RET*	Medullary thyroid cancer, pheochromocytoma
Neurofibromatosis type 1	Café au lait spots, axillary and inguinal freckling, and neurofibromas.	*NF1*	Optic gliomas, malignant peripherical nerve sheath tumours, breast
Birt-Hogg-Dubé	Skin fibrofolliculomas, spontaneous pneumothorax	*FLCN*	Renal

Cronkhite-Canada Syndrome is also characterized by gastrointestinal hamartomatous polyposis and is sometimes classified as a HPS. The syndrome is not considered to be hereditary but appears to be an autoimmune inflammatory condition [52].

### Genetic testing

The sometimes-difficult clinical and histopathological distinction between different HPS makes genetic testing important in the diagnostic approach to these patients. The development of *Next Generation Sequencing (NGS)* makes it possible to sequence DNA in a faster and less expensive way compared to traditional Sanger-sequencing. NGS holds a lot of possibilities for patients suspected of a polyposis syndrome, as it is now possible the test a panel of genes – or even all genes (exome-sequencing) -making an early and precise diagnosis. Nevertheless, it is not always possible – despite a clear histological and endoscopic diagnosis as well as a positive family history- to detect a germline mutation in any of the known genes. This indicates that new candidate genes are still to be discovered and that the genetic technology could be improved.

## Conclusion

Hamartomatous Polyposis Syndromes (HPS) are rare genetic syndromes characterized by the development of hamartomatous polyps in the gastrointestinal tract and an increased risk of cancer. Early diagnosis is important as affected patients and at-risk family members should be offered surveillance from an early age. The rapid development in genetics makes it possible to use genetic testing more often and thus genetic counselling should be a cornerstone in families with Hamartomatous Polyposis Syndromes.

## Abbreviations

GI-tract: Gastrointestinal tract; HPS: Hamartomatous Polyposis Syndromes; NGS: Next Generation Sequencing; JPS: Juvenile polyposis syndrome; PJS: Peutz-Jeghers syndrome; CS: Cowden Syndrome; BRRS: Bannayan-Riley-Ruvalcaba Syndrome; PS: Proteus syndrome; MEN2B: Multiple Endocrine Neoplasia type 2B; SIR: Standardised incidence ratio.

## Competing interests

The authors declare that they have no competing interests.

## Authors’ contributions

JAM: The main author who has conducted the main part of the literature search and written the first draft. QN: Contributed especially to the sections concerning surveillance and surgical interventions. BK: Contributed mainly to the sections concerning genetics and Next Generation Sequencing. NCB: Contributed especially to the section of Peutz-Jeghers Syndrome as well as providing pictures for the review. HTP: Contributed with histopathological descriptions and illustrations. OLB: Contributed especially to the part of Juvenile Polyposis syndrome, genetics and surveillance. All authors read and approved the final manuscript.
